# Supportive care needs after surgery in patients with breast cancer

**DOI:** 10.1007/s00520-024-08745-3

**Published:** 2024-08-07

**Authors:** Selda Rizalar, Elif Hamarat, Sonay Goktas

**Affiliations:** 1 Department of Surgical Nursing, Hamidiye Faculty of Nursing, University of Health Sciences, Istanbul, 34668 Turkey; 2 Hamidiye Health Sciences Institute, University of Health Sciences, Istanbul, Turkey

**Keywords:** Breast cancer, Cancer care, Supportive care, Nursing, Needs

## Abstract

**Purpose:**

This study aimed to determine supportive care needs and related factors after surgery in patients with breast cancer.

**Methods:**

This cross-sectional study was conducted with 98 breast cancer patients in a Training and Research Hospital in Istanbul between September 2022 and November 2023. The Personal Information Form and the Supportive Care Needs Survey Short Form Turkish version were used to collect data. One-way variance analysis, post hoc (Tukey, LSD), and *t*-test were used to analyze the data.

**Results:**

The total scale mean score for women who underwent surgery for breast cancer in the study was 83.95 22.97. Statistically significantly higher total scale scores were observed in younger women and those who received chemotherapy and radiotherapy than in others. The mean physical and daily living subscale scores of those who received chemotherapy and radiotherapy were higher than those who did not (*p* < .05). The psychology subscale mean scores of those who were young and unemployed were higher than the others (*p* < .05). The mean sexuality scores of those who were young, those with high education levels, and those who received chemotherapy were higher than the other groups (*p* < .05). Age factor affects SCN scores in women with breast cancer.

**Conclusion:**

Supportive care needs are higher among women with breast cancer who are younger and receive chemotherapy and radiotherapy. The physical needs of those who receive chemotherapy and radiotherapy, the psychological needs of those who are younger and unemployed, and the need for support regarding sexuality were greater among those who are younger and with higher education. Nurses should be aware of the specific needs of these disadvantaged groups and provide individualized holistic care.

## Introduction

Supportive care involves providing essential services for cancer patients’ physical, psychological, social, cognitive, and spiritual needs throughout the disease course [1, 2]. It encompasses activities and healthcare services that help the patient maximize the benefits of treatment and live with the effects of the disease as best as possible. Planning supportive care services for cancer patients should begin with identifying the patients’ priority needs [3].

The goal of supportive care is to enhance the patient’s physical comfort, support them psychosocially and spiritually, and maximize their well-being by addressing their information needs [4]. Effective communication with patients, informing and educating them, ensuring adherence to the treatment plan, assessing mental distress, and facilitating the patient’s participation in decisions throughout the cancer process are achieved through supportive care [4]. Supportive care is reported to increase survival and improve quality of life [5].

The trend in modern medicine is evolving from disease-based care to patient-centered care, where patients’ preferences and care needs are considered. Determining patient needs is essential for improving the quality of care [6]. Thus, care provided for each cancer patient should be tailored to their individual needs and coping mechanisms. Coping with cancer and adapting to its consequences are influenced by many factors, such as the individual’s perception of the disease, as well as socioeconomic status, education, presence of social supports, cultural characteristics, religion, and geographical location [7].

Khajoei et al.’s (2023) systematic review indicates that the most common needs among individuals with breast cancer are emotional and informational [6]. In the study of Chou et al. (2020), psychosocial needs ranked first in the list of unmet support needs of patients with breast cancer, followed by nutrition and patient care [8]. In the study conducted by Al Azri et al. (2022) with Omani women diagnosed with breast cancer, the needs of the patients were primarily related to the health system and information, followed by patient care and support [9]. In the study of Elsous et al. (2023), the most frequently reported needs of women with breast cancer were psychological, followed by needs related to the health system and information, physical and daily living [10].

In Turkey, several studies have explored the care needs of various cancer types; however, breast cancer samples have not been specifically investigated. In a survey by Çelik (2021), the needs priorities of cancer patients were listed as psychological and needs to be related to health care and information, daily living, and sexuality [4]. Erarslan’s (2021) study reported that lung cancer patients had the highest supportive care needs in the psychological and health services and information areas [11]. In the study by Yücel et al. (2022) examining oncological surgery patients’ supportive care needs, support was needed most in physical and daily living activities [12]. Most patients with breast cancer undergo surgery soon after diagnosis and receive other treatments before or after surgery. Identifying supportive care needs during and after the treatment process can lead to a better understanding of cancer experiences and prevent unwanted situations. Moreover, targeted intervention approaches can be planned to ensure individuals can go through the treatment process more comfortably. In this study, we examined the supportive care needs and affecting factors in breast cancer patients who underwent surgery.

Our research questions were as follows:

- What is the level of supportive care needs?
- Is there an association between demographic and clinical characteristics and supportive care needs?

## Method

### Study design and setting

This study employed a cross-sectional design. “The Strengthening the Reporting of Observational Studies in Epidemiology (STROBE) Checklist” was used as a guideline [13].

The research was conducted at a General Surgery Department’s outpatient breast diseases clinic in a Training and Research Hospital in Istanbul.

### Participants and procedure

The study population included women who had undergone surgical treatment for breast cancer and had been followed in the specified hospital in the last 6 months. The number of patients followed up during this period was 130. The required sample size was calculated using the formula for known populations in stratified sampling (Nt^2^pq/(N-1) + t^2^pq) and determined to be at least 98 patients [14]. The sample consisted of women who applied to the breast outpatient clinic between September 2022 and November 2023 and underwent surgery with a diagnosis of breast cancer.

Inclusion criteria were being aged 18 years and above, conscious, able to communicate, literate in Turkish, having applied to the breast outpatient clinic and undergone surgery with the diagnosis of breast cancer, initiated breast cancer treatment with at least 1 month elapsed since the start, and voluntary participation in the study.

Individuals who did not agree to participate in the research, answered the survey incompletely, and whose general condition was not suitable for answering the survey were excluded from the study. The data of 102 patients who volunteered to participate in the study and met the inclusion criteria were collected, and the data of 98 breast cancer patients were evaluated.

#### Ethics

By complying with the Principles of the Declaration of Helsinki, written permission was obtained from the Hamidiye Scientific Research Ethics Committee of the University of Health Sciences (Date:25.03.2022 Decision Number:10/6) and the Scientific Committee of the Training and Research Hospital where the research was conducted before collecting the data. The individuals included in the study were provided with necessary explanations, and written consent was obtained from those who agreed to participate.

### Measurements

Data were collected with the Personal Information Form and the “Supportive Care Needs Scale—Short Form (SCNS-SF 29Tr).

#### Personal information form

The form, prepared by the researchers based on a literature review, included questions covering demographic and clinical characteristics [4, 5, 15].

#### The short-form supportive care needs survey questionnaire in Turkish (SCNS-SF 29.^TR^)

SCNS-SF 29, designed by Boyes, Girgis and Lecathelinais (2009) to evaluate the care needs of cancer patients [16], and whose Turkish validity and reliability study was conducted by Özbayır et al. (2017), was used in data collection. The Cronbach α reliability coefficient of the scale was found to be 0.93 in the study by Özbayır et al. The five-point Likert-type scale contains 29 items in five subscales: psychology, health system and information, physical and daily living, patient care and support, and sexuality. Scores between 29 and 145 can be obtained from the scale. A higher total score means a greater perceived need for support.

### Data collection procedure

Data were obtained by face-to-face interview method, planned in a way that would not disrupt the treatment plan. Participants were provided with information about the purpose and significance of the study and invited to take part. Data collection tools were administered to those who agreed to participate after completing the informed consent form. Completing the study forms took approximately 15–20 min for one interview. Medical records were checked to update the participant’s cancer type, stage, and medical treatment information. The participants were assured that only the researchers would have access to their data and that their personal information would remain confidential.

### Data analysis

The data were analyzed by IBM SPSS Statistics for Windows, Version 22.0. Armonk, NY: IBM Corp. Frequency, percentage, mean, and standard deviation were used to determine descriptive characteristics. Kurtosis and Skewness values were examined to determine whether the research variables showed normal distribution. Since the data showed normal distribution, parametric methods were employed for the analysis. Independent groups t-test, one-way analysis of variance (ANOVA) and post hoc (Tukey, LSD) analyses were performed to examine the differences in scale levels according to the descriptive characteristics of the patients. Multivariate regression analysis was performed to determine the variables affecting SCN. The difference was considered statistically significant if the obtained *p*-value was less than 0.05.

## Results

The findings related to the descriptive characteristics of the patients are presented in Table 1.

**Table 1:** Sociodemographics and clinical characteristics of breast cancer patients

Characteristics frequency (*n*) percentage (%)		
Age group		
18–44 45–54 55 and above	29 40 29	29.6 40.8 29.6
Marital status		
Married Single	74 24	75.5 24.5
Education level		
Primary education High school University	51 29 18	52.0 29.6 18.4
Employment		
Employed Unemployed	33 65	33.7 66.3
Income level		
Income is less than expenses Income equals to expenses Income is more than expenses	43 55 0	43.9 56.1 0.0
Person(s) she lives together at home		
Alone Spouse Spouse and children Children Parents	5 15 54 17 7	5.1 15.3 55.1 17.3 7.1
Health assurance/insurance		
Yes No	92 6	93.9 6.1
Smoking habit		
Yes No	23 75	23.5 76.5
Alcohol habit		
Yes No	1 97	1.0 99.0
Chronic condition		
Yes No	36 62	36.7 63.3
Regular medication		
Yes No	44 54	44.9 55.1
Type of surgery		
BCS 63 64.3		
Mastectomy 35 35.7		
The time since the first treatment		
2 months 3–6 months 7–11 months 1–2 years 2 years and more	14 30 27 16 11	14.3 30.6 27.6 16.3 11.2
Previous surgery		
Yes No	97 1	99.0 1.0
Chemotherapy		
Yes No	64 34	65.3 34.7
Radiotherapy		
Yes No	39 59	39.8 60.2
Hormonotherapy		
Yes No	18 80	18.4 81.6
The presence of someone providing care support		
Yes No	66 32	67.3 32.7
One More than one	55 11	83.3 16.7
The person providing care support		
Spouse Child Parent More than one	26 22 7 11	39.4 33.3 10.6 16.7
Stage		
Early (1–2) Advanced (3–4)	58 40	59.2 40.8

29.6% of the participants were 29.6% in the 18–44 age group, 40.8% in the 45–54 age group, and 29.6% in the 55 and above age group. 75.5% were married, and 52% were primary school graduates. 33.7% were employed. 43.9% had low family income, and 56.1% had average family income. 55.1% lived with their spouse and children, 17.3% with their children, 15.3% with their spouse, 7.1% with their parents, and 5.1% alone. 93.9% had health insurance. Smoking was present in 23.5%, alcohol consumption in 1%, and 36.7% had additional chronic diseases, while 44.9% used medication regularly.

Considering the type of surgical treatment, 64.3% of the women had undergone BCS (Breast Conserving Surgery) and 35.7% had a mastectomy.

The duration of treatment for the patients was 2 months in 14.3%, 3–6 months in 30.6%, and 7–11 months in 27.6%. Chemotherapy was applied to 65.3%, radiotherapy to 39.8%, and hormonotherapy to 18.4%. 67.3% of the women had someone to support them with their care, with 39.4% being their spouse and 33.3% being their child. 59.2% were in the early, and 40.8% were in the advanced stage of cancer.

The patients’ mean scores from the total supportive care needs scale and subscales are shown in Table 2.

**Table 2:** Supportive care needs scale mean scores of patients with breast cancer (*N* = 98)

Supportive care M (SD) min. –max Cr alpha needs scale			
Total	83.95 (22.97)	39–138	0.933
Subscales			
Physical and daily living Psychological Sexuality Health system and information Patient care and support	14.63 (5.48) 26,52 (8.88) 6.41 (3.44) 21.63 (7.51) 14.75 (5.02)	5–25 10–45 3–15 7–35 5–25	0.896 0.908 0.912 0.923 0.903

SCN scale mean scores by the demographic and clinical characteristics of the women included in the study are given in Table 3. The age variable produced statistically significant differences in both the overall SCNS total scores and subscale scores: SCNS total scores of those in the age group 18–44 were higher than the scores of other groups (*p* < 0.05). Also, those in the age group 18–44 had higher psychology subscale scores than the other groups (*p* < 0.05). Additionally, the sexuality subscale scores of the 18–44 age group patients were higher than the others (*p* < 0.05).

**Table 3:** Distribution of supportive care needs scale mean scores according to demographic and clinical characteristics of patients with breast cancer

Descriptive features	*n*	Supportive care needs total Mean ± SD	Physical and daily living Mean ± SD	Psychological Mean ± SD	Sexuality Mean ± SD	Health system and information Mean ± SD	Patient care and support Mean ± SD
Age							
18–44	29	93.10 ± 20.31	16.34 ± 5.97	30.03 ± 6.53	8.17 ± 3.47	22.93 ± 8.18	15.62 ± 5.10
45–54	40	81.20 ± 23.31	14.45 ± 5.48	25.80 ± 9.13	5.95 ± 3.35	20.65 ± 7.46	14.35 ± 5.09
55 and above	29	78.59 ± 23.04	13.17 ± 4.61	24.00 ± 9.72	5.28 ± 2.90	21.69 ± 6.92	14.45 ± 4.90
*F* =		*3.558*	*2.546*	*3.773*	*6.377*	*0.773*	*0.611*
*p* =		***0.032***	*0.084*	***0.027***	***0.003***	*0.465*	*0.545*
PostHoc =		*1* > *2, 1* > *3 (p* < *0.05)*		*1* > *2, 1* > *3 (p* < *0.05)*	*1* > *2, 1* > *3 (p* < *0.05)*		
Medeni Durum							
Married	74	84.18 ± 23.55	14.31 ± 5.56	26.76 ± 9.32	6.61 ± 3.49	21.81 ± 7.38	14.69 ± 4.78
Single	24	83.25 ± 21.56	15.62 ± 5.19	25.79 ± 7.48	5.79 ± 3.24	21.08 ± 8.04	14.96 ± 5.81
*t* =		*0.171*	* − 1.021*	*0.461*	*1.011*	*0.411*	* − 0.227*
*p* =		*0.865*	*0.310*	*0.646*	*0.315*	*0.682*	*0.821*
Education level							
Primary education	51	81.04 ± 24.76	14.27 ± 5.32	25.16 ± 9.15	5.37 ± 3.16	21.78 ± 7.92	14.45 ± 5.27
High school	29	88.00 ± 22.15	15.48 ± 6.35	28.38 ± 8.95	7.69 ± 3.47	21.59 ± 7.97	14.86 ± 5.10
University	18	85.67 ± 18.51	14.28 ± 4.47	27.39 ± 7.72	7.28 ± 3.37	21.28 ± 5.69	15.44 ± 4.29
*F* =		*0.909*	*0.490*	*1.331*	*5.337*	*0.030*	*0.266*
*p* =		*0.407*	*0.614*	*0.269*	***0.006***	*0.970*	*0.767*
PostHoc =					*2* > *1, 3* > *1 (p* < *0.05)*		
Employment							
Employed	33	79.73 ± 22.98	14.76 ± 5.24	24.06 ± 7.97	6.97 ± 3.23	19.58 ± 6.68	14.36 ± 5.29
Unemployed	65	86.09 ± 22.84	14.57 ± 5.64	27.77 ± 9.12	6.12 ± 3.53	22.68 ± 7.74	14.95 ± 4.90
*t* =		* − 1.301*	*0.160*	* − 1.983*	*1.153*	* − 1.960*	* − 0.548*
*p* =		*0.196*	*0.873*	***0.050***	*0.252*	*0.053*	*0.585*
Income level							
Income is less than expenses	43	87.14 ± 23.56	15.21 ± 5.63	28.07 ± 8.58	6.60 ± 3.55	22.60 ± 7.74	14.65 ± 4.83
Income equals to expenses	55	81.45 ± 22.39	14.18 ± 5.37	25.31 ± 9.00	6.25 ± 3.37	20.87 ± 7.31	14.84 ± 5.21
*t* =		*1.219*	*0.920*	*1.538*	*0.498*	*1.134*	* − 0.180*
*p* =		*0.226*	*0.360*	*0.127*	*0.620*	*0.259*	*0.857*
Person(s) she lives together at home							
Alone	5	99.20 ± 27.63	15.80 ± 7.05	29.60 ± 7.40	8.40 ± 5.27	26.20 ± 8.93	19.20 ± 6.02
Spouse	15	79.07 ± 23.76	14.07 ± 4.85	27.40 ± 9.55	5.20 ± 2.68	19.53 ± 7.10	12.87 ± 4.82
Spouse and children	54	84.54 ± 23.00	14.39 ± 5.73	26.11 ± 9.40	7.06 ± 3.53	22.00 ± 7.29	14.98 ± 4.62
Children	17	84.47 ± 20.93	16.12 ± 5.07	25.23 ± 7.85	5.06 ± 2.77	22.82 ± 7.56	15.23 ± 5.32
Parents	7	77.71 ± 23.62	13.29 ± 5.25	28.71 ± 7.72	5.86 ± 3.08	17.14 ± 7.88	12.71 ± 5.76
*F* =		*0.855*	*0.531*	*0.401*	*2.157*	*1.554*	*1.939*
*p* =		*0.494*	*0.713*	*0.807*	*0.080*	*0.193*	*0.110*
Health assurance/insurance							
Yes	92	84.03 ± 23.11	14.53 ± 5.46	26.27 ± 8.93	6.46 ± 3.49	21.90 ± 7.45	14.87 ± 5.04
No	6	82.67 ± 22.66	16.17 ± 6.14	30.33 ± 7.74	5.67 ± 2.73	17.50 ± 7.82	13.00 ± 4.69
*t* =		*0.140*	* − 0.706*	* − 1.086*	*0.543*	*1.398*	*0.883*
*p* =		*0.889*	*0.482*	*0.280*	*0.588*	*0.165*	*0.379*
Smoking habit							
Yes	23	81.61 ± 22.39	14.52 ± 5.98	26.04 ± 8.37	6.96 ± 3.64	20.22 ± 7.91	13.87 ± 5.25
No	75	84.67 ± 23.25	14.67 ± 5.36	26.67 ± 9.08	6.24 ± 3.38	22.07 ± 7.39	15.03 ± 4.95
*t* =		* − 0.557*	* − 0.110*	* − 0.293*	*0.873*	* − 1.033*	* − 0.967*
*p* =		*0.579*	*0.912*	*0.770*	*0.385*	*0.304*	*0.336*
Presence of a chronic condition							
Yes	36	83.08 ± 23.71	14.22 ± 5.29	26.11 ± 9.19	5.472 ± 3.32	22.28 ± 7.15	15.00 ± 4.61
No	62	84.45 ± 22.71	14.87 ± 5.62	26.76 ± 8.76	6.952 ± 3.42	21.26 ± 7.74	14.61 ± 5.27
*t* =		* − 0.283*	* − 0.563*	* − 0.346*	* − 2.088*	*0.646*	*0.366*
*p* =		*0.778*	*0.575*	*0.730*	***0.039***	*0.520*	*0.715*
Regular medication							
Yes	44	87.25 ± 23.57	14.95 ± 5.10	27.34 ± 8.91	6.07 ± 3.62	23.16 ± 7.28	15.73 ± 4.85
No	54	81.26 ± 22.33	14.37 ± 5.80	25.85 ± 8.88	6.68 ± 3.29	20.39 ± 7.53	13.96 ± 5.06
*t* =		*1.289*	*0.523*	*0.824*	* − 0.882*	*1.838*	*1.749*
*p* =		*0.201*	*0.602*	*0.412*	*0.380*	*0.069*	*0.084*
Type of surgery							
BCS	63	83.90 ± 25.10	14.49 ± 5.70	27.03 ± 9.35	6.35 ± 3.50	21.30 ± 7.74	14.73 ± 5.11
Mastectomy	35	84.03 ± 18.89	14.89 ± 5.13	25.60 ± 8.01	6.51 ± 3.38	22.23 ± 7.15	14.80 ± 4.93
		* − 0.025*	* − 0.339*	*0.763*	* − 0.227*	* − 0.583*	* − 0.066*
		*0.978*	*0.735*	*0.447*	*0.821*	*0.561*	*0.948*
The time elapsed since the first treatment							
2 months	14	77.29 ± 20.80	11.57 ± 5.39	24.00 ± 9.01	6.29 ± 2.81	20.71 ± 6.32	14.71 ± 4.41
3–6 months	30	81.33 ± 21.14	13.93 ± 5.98	25.93 ± 8.10	5.57 ± 3.40	21.93 ± 7.24	13.97 ± 5.01
7–11 months	27	90.48 ± 25.56	16.22 ± 4.47	28.63 ± 9.52	6.93 ± 3.54	22.89 ± 7.87	15.81 ± 5.00
1–2 years	16	85.44 ± 24.05	15.44 ± 5.66	27.00 ± 9.89	7.06 ± 3.85	21.19 ± 7.67	14.75 ± 5.11
2 years and above	11	81.36 ± 22.02	15.36 ± 5.20	25.45 ± 7.95	6.64 ± 3.53	19.55 ± 9.12	14.36 ± 6.09
*F* =		*0.989*	*1.996*	*0.738*	*0.755*	*0.469*	*0.492*
*p* =		*0.418*	*0.101*	*0.568*	*0.557*	*0.758*	*0.741*
Chemotherapy							
Yes	64	87.94 ± 24.02	16.19 ± 5.19	27.36 ± 8.78	6.92 ± 3.63	22.22 ± 7.87	15.25 ± 5.07
No	34	76.44 ± 18.98	11.71 ± 4.83	24.94 ± 8.99	5.44 ± 2.84	20.53 ± 6.76	13.82 ± 4.86
*t* =		*2.416*	*4.166*	*1.287*	*2.062*	*1.060*	*1.345*
*p* =		***0.018***	***0.000***	*0.201*	***0.029***	*0.292*	*0.182*
Radiotherapy							
Yes	39	90.00 ± 24.42	16.54 ± 5.14	27.08 ± 9.61	7.13 ± 3.59	23.33 ± 7.51	15.92 ± 5.10
No	59	79.95 ± 21.23	13.37 ± 5.37	26.15 ± 8.43	5.93 ± 3.28	20.51 ± 7.36	13.98 ± 4.85
*t* =		*2.160*	*2.904*	*0.502*	*1.701*	*1.845*	*1.898*
*p* =		***0.033***	***0.005***	*0.617*	*0.092*	*0.068*	*0.061*
Hormonotherapy							
Yes	18	85.44 ± 25.02	14.06 ± 5.33	25.89 ± 9.84	6.67 ± 3.22	23.11 ± 7.49	15.72 ± 4.94
No	80	83.61 ± 22.64	14.76 ± 5.54	26.66 ± 8.71	6.35 ± 3.50	21.30 ± 7.52	14.54 ± 5.04
*t* =		*0.304*	* − 0.493*	* − 0.332*	*0.351*	*0.924*	*0.904*
*p* =		*0.762*	*0.623*	*0.740*	*0.726*	*0.358*	*0.368*
The presence of someone providing care support							
Yes	66	81.38 ± 22.99	14.08 ± 5.56	25.73 ± 9.34	6.03 ± 3.36	21.14 ± 7.11	14.409 ± 4.75
No	32	89.25 ± 22.34	15.78 ± 5.20	28.16 ± 7.73	7.19 ± 3.52	22.66 ± 8.30	15.469 ± 5.55
*t* =		* − 1.604*	* − 1.453*	* − 1.274*	* − 1.574*	* − 0.939*	* − 0.980*
*p* =		*0.112*	*0.149*	*0.206*	*0.119*	*0.350*	*0.330*
Number of persons supporting care							
One	55	81.42 ± 23.84	13.82 ± 5.64	26.04 ± 9.37	6.15 ± 3.48	21.07 ± 7.36	14.35 ± 4.96
More than one	11	81.18 ± 19.22	15.36 ± 5.22	24.18 ± 9.46	5.45 ± 2.73	21.45 ± 6.01	14.73 ± 3.69
*t* =		*0.031*	* − 0.839*	*0.598*	*0.620*	* − 0.161*	* − 0.242*
*p* =		*0.975*	*0.404*	*0.552*	*0.538*	*0.872*	*0.810*
The person providing care support							
Spouse	26	85.46 ± 23.81	15.23 ± 5.17	28.11 ± 9.28	6.61 ± 3.53	20.96 ± 7.07	14.54 ± 4.54
Child	22	76.45 ± 21.31	13.36 ± 5.61	23.77 ± 9.45	4.91 ± 2.69	20.68 ± 7.05	13.73 ± 5.14
Parent	7	80.86 ± 31.62	11.00 ± 6.71	26.86 ± 9.15	7.29 ± 4.79	21.43 ± 10.15	14.29 ± 6.58
More than one	11	81.91 ± 19.24	14.73 ± 5.42	23.27 ± 9.11	6.09 ± 2.91	22.27 ± 5.95	15.55 ± 3.27
*F* =		*0.601*	*1.274*	*1.185*	*1.434*	*0.127*	*0.358*
*p* =		*0.617*	*0.291*	*0.323*	*0.241*	*0.943*	*0.783*
Stage							
Early (1–2)	58	82.72 ± 22.12	14.05 ± 5.38	26.29 ± 8.78	6.64 ± 3.49	21.19 ± 7.48	14.55 ± 5.08
Advanced (3–4)	40	85.72 ± 24.33	15.47 ± 5.57	26.85 ± 9.13	6.07 ± 3.38	22.27 ± 7.61	15.05 ± 4.98
*t* =		* − 0.634*	* − 1.268*	* − 0.304*	*0.795*	* − 0.701*	* − 0.481*
*p* =		*0.528*	*0.208*	*0.762*	*0.429*	*0.485*	*0.632*

*F*, ANOVA test; *t*, independent groups *T*-test; *PostHoc*, LSD

The patients did not differ significantly in SCNS total and subscale scores by marital status (*p* > 0.05).

By education level, the participants differed only in sexuality scores, with the high school and university graduates having higher scores than primary school graduates (*p* < 0.05).

By employment status, the only difference was in the psychology subscale scores, where the unemployed participants had higher psychology mean scores than those who were unemployed (*p* < 0.05).

By income level, the SCNS total scores of patients with low income (87.14 ± 23.56) were higher than those with average income (81.45 ± 22.39). However, the difference between SCNS total and subscale mean scores was not statistically significant (*p* > 0.05).

By health insurance status, no statistically significant difference was found in SCNS total and subscale mean scores (*p* > 0.05).

When the SCNS total and subscale mean scores were compared considering the people with whom the participants lived, those living alone had higher scores, especially from the total SCNS and patient care and support subscales. However, the difference was not significant (*p* > 0.05).

By the presence of a chronic condition, a statistically significant difference was found only between the sexuality subscale scores (*p* < 0.05), with those without a chronic condition having higher scores.

The participants did not differ significantly in the SCNS total and subscale mean scores by smoking habit and regular medication use (*p* > 0.05).

SCNS total scores of those who had mastectomy (84.03 ± 18.89) were higher than those who had BCS (83.90 ± 25.1), but the difference was not statistically significant (*p* > 0.05).

By the time since the initial treatment, those with 2 months elapsed since the initial treatment had the lowest score (77.29 ± 20.80), while those with 3–6 months, 7–11 months, 1–2 years, and more than two years since the initial treatment scored 81.33 ± 21.14, 90.48 ± 25.56 (highest), 85.44 ± 24.05, and 81.36 ± 22.02, respectively. However, there was no statistically significant difference in SCNS total and subscale scores based on the time since the initial treatment (*p* > 0.05).

SCNS total, physical and daily living, and sexuality subscale scores of patients who received chemotherapy were statistically significantly higher than those who did not (*p* < 0.05). SCNS total and physical and daily living scores of those who received radiotherapy were significantly higher than those who did not (*p* < 0.05).

SCNS total scores of women with advanced cancer (85.72 ± 24.33) were higher than those with early-stage cancer (82.72 ± 22.12). However, the difference between SCNS total and subscale mean scores was not statistically significant (*p* > 0.05).

There was no significant difference between the SCNS total and subscale scale scores depending on whether there was a person supporting home care, the number of support persons, or the identity of the supporting person (*p* > 0.05).

Multivariate regression analysis was performed to determine the variables affecting supportive care needs (Table 4). Variables that were significant in pairwise comparisons were included in the model. Categorical independent variables were changed as dummy variables and included in the analysis.

**Table 4:** Multipl regression associations of SCNS total scores with age and receiving chemotherapy or radiotherapy

İndependent variables	*B*	SE	*β*	*t*	*p*	%95 conf. interval	
						Lower bound	Upper bound
(Constant)	63.122	18.886		3.342	0.001	25.618	100.626
Age (Ref: 18–44)							
*45*–*54*	* − *12.651	5.446	* − *0.272	2.323	**0**.**022**	1.837	23.464
*55 and above*	* − *11.914	5.909	* − *0.238	2.016	**0**.**047**	0.179	23.648
Receiving chemotherapy	* − *5.723	5.683	* − *0.119	* − *1.007	0.317	* − *17.008	5.562
Receiving radioterapy	* − *7.431	5.532	* − *0.159	* − *1.343	0.182	* − *18.415	3.554

*B*, unstandardized coefficients; *β*, standardized coefficient; *SE*, standard error

^*^Dependent variable = SCNS total *R* = 0.355; *R*^2^ = 0.089; *F* = 3.357;* p* = 0.013; Durbin Watson value = 2.489

Regression analysis to determine the cause and effect relationship between age, receiving chemotherapy or radiotherapy and supportive care needs was significant (*F* = 3.357; *p* = 0.013 < 0.05). The total change in the level of SCN was explained by age, receiving chemotherapy and radiotherapy at a rate of 8.9% (R2 = 0.089).

According to our findings, being in the 18–44 age group increases the level of supportive care needs compared to being in the 45–54 age group (*β* =  − 0.272). At the same time, being between 18 and 44 years of age increases supportive care requirements compared to being 55 and over (*β* =  − 0.238). According to the advanced test findings, chemotherapy did not affect SCN (*p* = 0.317 > 0.05) and radiotherapy did not affect SCN too (*p* = 0.182 > 0.05).

## Discussion

This study is the first investigation of supportive care needs conducted exclusively in the breast cancer population in Turkey. In our study, the majority of women with breast cancer were in the 45–54 age group, had primary school education, were married, were unemployed, and had an average level of family income.

We determined the SCNS total score in breast cancer patients as 83.95 ± 22.97 (39–138). Similar levels were found in the study by Uslu et al. (2023) in women with gynecological cancer, with a total SCNS score of 84.93 ± 18.86 (36–127) [17]. Another study conducted in Turkey also including stage IV patients, where one-third of the participants had breast cancer with a mean time since diagnosis of 1–6 months, found a slightly higher SCNS total score (101.19 ± 33.97) [4].

The perception of a person diagnosed with cancer about SCN may vary depending on age, type and stage of cancer and throughout the cancer process [18]. According to our findings, young women’s psychological and sexual support needs were higher than middle-aged and older women. According to Fan et al.’s review, psychological needs ranked second on the list of unmet supportive needs in women with breast cancer [19]. In the study of Abdollahzadeh et al. (2014), young women with breast cancer had more unmet needs in all areas [20]. In a study involving patients diagnosed with gastrointestinal and breast cancer, approximately half of all the needs of the patients were not met, and the unmet needs were primarily informational and psychosocial [21]. It has been noted that one-third of young women undergoing breast cancer treatment begin survivorship with the Distress Thermometer (DT) in the early stages, and needs decrease over time [22].

Studies have reported that young female patients need supportive care in many areas, especially in sexual function [23, 24]. Besides, surgical treatment and chemotherapy that women with breast cancer receive seem to affect body image and femininity role, highlighting the needs related to sexuality [25]. The overall prevalence of sexual dysfunction in female cancer survivors ranges from 16.7–67% [26]. Sexual problems commonly seen in breast cancer due to surgery and other treatments reduce women’s quality of life [27]. Furthermore, unmet SCN in cancer patients may have negative consequences. Indeed, Uslu et al.’s (2024) study on women with gynecological cancer found unmet psychological and sexual needs as negative determinants of acceptance of illness [17]. Sexuality considerations in breast cancer with substantial impacts on overall health and quality of life remain an unmet need.

In this study, women with high school and university education levels had higher perceptions of SCN in sexuality than those with primary school education. Women with higher education levels appear to recognize and express their needs regarding sexuality better. Those with lower education levels may be more influenced by societal gender stereotypes and find it more difficult to acknowledge and communicate their sexual needs. Still considered taboo in some cultures [28, 29], sexuality also tends to be ignored in Turkish culture [30]. This tendency may be particularly true in uneducated women.

The total mean SCN score of the married women in our study was slightly higher than that of single women, but the difference was not statistically significant. Studies have reported contradicting findings regarding the effect of marital status on SCN in breast cancer patients. In Chae’s (2019) study, women with breast cancer without spouses were found to have more needs in areas of information and education, psychological problems, healthcare personnel and hospital service [31]. In the study conducted by Abdollahzadeh et al. (2014) with women who completed their first treatment in Iran, married women were found to have more unmet supportive care needs related to sexuality [20]. Women with breast cancer should have their supportive care needs taken into account by health professionals regardless of their marital status.

In our research, the psychological care needs of unemployed women were higher than those who were employed. Our findings suggest that being employed can be beneficial in meeting women’s psychological needs. Gebresillassie et al.’s (2020) study reported higher physical SCN in unemployed individuals, and Chae’s (2019) study indicated greater educational and knowledge needs among the unemployed [31, 32]. Also, higher levels of acceptance of illness have been reported for working women [33]. Breast cancer survivors returning to their careers may find it easier to fulfil both their physical and psychological needs.

Income level, which generally influences individuals’ quality of life, can also affect the healthcare service and quality of care received during illness. In our study, the SCN scores of low-income women were slightly higher than those with average income. In disadvantaged groups with insufficient income, particularly in rural areas, there are known inequalities in meeting SCN [19, 34], although a study reported that individuals with higher incomes had more SCN [32]. Therefore, SCn in breast cancer survivors should be considered irrespective of their income level.

The women with breast cancer with comorbidities in our study had lower sexual need scores. Individuals with additional health-related limitations may prioritize their sexual needs less due to the effects of comorbidities, potentially leading to sexual dysfunction. Indeed, Cayan et al.’s (2004) study identified a higher prevalence of sexual dysfunction in individuals with chronic diseases, including cancer [35]. Moreover, lower sexual adjustment scores have been reported for breast cancer patients with chronic health issues [36]. The sexual needs of women with breast cancer should be recognized and addressed by the nurse.

The SCN total score of the patients living alone at home (99.200 ± 27.635) was higher than that of those living with their spouse (79.067 ± 23.762). The SCN score of women who did not have a care support person (89.25 ± 22.34) also was higher than those who did (81.38 ± 22.99). Patients with poor support resources are expected to have greater care needs. Social support is recognized as a significant factor affecting the well-being of breast cancer patients, and it may even impact survival [31]. Survivors without a spouse have greater needs as they have to rely solely on medical personnel. In the study by Chae et al. (2019), survivors without a spouse had more unmet needs in many areas [31]. Living alone is a forerunner to unmet needs in patient care [23]. Klungrit et al.’s study (2019) reported that women needed assistance from both healthcare professionals and family members to alleviate physical symptoms and treatment side effects [37]. It can be said that individuals living alone need more supportive care.

It is known that the disease process of women with breast cancer who have undergone mastectomy is more complex and problematic than those who have had BCS. In our study, the needs of women who underwent mastectomy and those who underwent BCS were similar. This finding reveals that women with breast cancer who have undergone BCS have as many needs as those who have had mastectomy. The needs of women undergoing BCS should also be well recognized and addressed without underestimating them.

In a study involving different cancer survivors, higher SCN was found in the early post-diagnosis period [8, 38]. It has been stated that SCNs in breast cancer vary at different stages of the treatment process [39], mostly occurring in the early stages of survival [5]. This is because the symptom burden and treatment side effects are more severe in this period [31, 38, 40]. It is also known that patient’s needs decrease over time. Our study did not find a statistically significant difference in total SCN scores based on the time elapsed since the initial treatment. However, survivors, especially those 7–11 months after their first treatment, exhibited higher needs compared to others. This period corresponds to the end of the patients’ medical treatment process after surgery and means reduced contact with healthcare professionals, necessitating readjusting to their previous social life. Literature suggests that patients perceive variations, especially in healthcare professional support, after the completion of treatment, expressing concerns about home care [34]. A cohort study with outpatient cancer patients associated a longer time since diagnosis with increased care needs [41]. Our findings regarding the time from diagnosis are consistent with the literature.

Needs not only increase or decrease depending on the early or advanced stage of the disease but can also change in their nature. A retrospective study conducted in Taiwan with cancer patients reported that newly diagnosed patients had higher information needs while relapsing and terminal patients had greater psychosocial needs [8].

There are also studies emphasizing that needs mainly arise during the treatment phase [42]. In our study, the total SCNs and physical needs of those who received chemotherapy and radiotherapy were higher than those who did not. In the study of Doğan et al., the psychological states of individuals diagnosed with cancer were more affected during the chemotherapy process [25]. Additionally, the more therapies combined, the higher the perceived SCN levels [31, 40]. Different types of surgery and treatments create different care needs. Needs progressively escalate from surgery alone to a combination of surgery and radiotherapy and even further when surgery, radiotherapy, and chemotherapy are combined [23]. Additionally, in our study, the sexual needs of those who received chemotherapy were higher than those who did not. This can be explained by the negative impact of chemotherapy’s effects and side effects on an individual’s body image and self-esteem, leading to psychosocial issues.

In our study, the SCN total scores of women with advanced cancer were higher than those with early-stage cancer, but no statistically significant difference was found. In the literature, SCN has been reported to be higher in patients with advanced breast cancer [31, 40, 43]. Fan et al. (2023) found that SCNs were more common when the cancer was more advanced [19]. Our finding may be attributed to the increased needs of patients in advanced stages of cancer as they experience more intense symptoms.

Having high levels of SCN can increase the fear of cancer recurrence and result in difficulties in performing daily life activities [40]. Unmet needs can reduce treatment adherence, leading to physical and psychological distress. It can also decrease survival rates and increase healthcare costs [18]. Cancer survivors who report unmet needs are reported to have a higher risk of emergency department visits and hospitalisations [41]. Therefore, SCN should first be diagnosed systematically and treated promptly without delay.

### Limitations

The sample for this study was obtained from a hospital in a metropolitan city center. The hospital primarily treats patients residing in the city center. No assessment has been made regarding the situation of women in rural areas.

## Conclusion

This study makes a significant contribution to the literature by identifying the supportive care needs (SCN) and influencing factors in women with breast cancer. The research demonstrated that Turkish breast cancer survivors under follow-up after surgical treatment have average-level supportive care needs. It was observed that the younger age group, those with higher education, and women undergoing chemotherapy or radiotherapy had more SCNs. Age factor affects supportive care needs in women with breast cancer. The physical needs of those receiving chemotherapy and radiotherapy, the psychological needs of younger and non-working women, and the sexual needs of younger and more educated women were found to be higher. Nurses who spend the most time with patients should evaluate and support patients holistically regarding their supportive care needs. Nurses should perform these actions in cooperation with the patient and families, employing a supportive attitude. They must establish a relationship based on trust, develop effective communication, and organize interventions tailored to the patient’s condition.

## Data Availability

No datasets were generated or analysed during the current study.
